# Interactions of β-Conglycinin (7S) with Different Phenolic Acids—Impact on Structural Characteristics and Proteolytic Degradation of Proteins

**DOI:** 10.3390/ijms17101671

**Published:** 2016-10-02

**Authors:** Jing Gan, Hao Chen, Jiyuan Liu, Yongquan Wang, Satoru Nirasawa, Yongqiang Cheng

**Affiliations:** 1 Beijing Key Laboratory of Functional Food from Plant Resources, College of Food Science and Nutritional Engineering, China Agricultural University, Beijing 100083, China; ganjing@cau.edu.cn (J.G.); haochen@cau.edu.cn (H.C.); liujiyuan@cau.edu.cn (J.L.); wangyq@cau.edu.cn (Y.W.); 2 Japan International Research Center for Agricultural Sciences, Enzyme Laboratory, Tsukuba 305-8686, Japan; stnirasa@affrc.go.jp

**Keywords:** *p*-coumalic acid, caffeic acid, gallic acid, chlorogenic acid, β-Conglycinin, 7S, interaction

## Abstract

*p*-Coumalic acid (PCA), caffeic acid (CA), gallic acid (GA) and chlorogenic acid (CGA) are the major phenolic acids that co-exist with soy protein components in foodstuffs. Surprisingly, there are only a handful of reports that describe their interaction with β-Conglycinin (7S), a major soy protein. In this report, we investigated the interaction between phenolic acids and soy protein 7S and observed an interaction between each of these phenolic acids and soy protein 7S, which was carried out by binding. Further analysis revealed that the binding activity of the phenolic acids was structure dependent. Here, the binding affinity of CA and GA towards 7S was found to be stronger than that of PCA, because CA and GA have one more hydroxyl group. Interestingly, the binding of phenolic acids with soy protein 7S did not affect protein digestion by pepsin and trypsin. These findings aid our understanding of the relationship between different phenolic acids and proteins in complex food systems.

## 1. Introduction

Soy proteins are plant macromolecules that have attracted significant attention in Western countries in recent years because their special elements relative to the animal proteins (e.g., rich in lysine and other essential amino acids) [1], and thus represent a substitute to meat products. β-Conglycinin (7S), as one of the major soy proteins, contains three subunits (α’, α and β) [2] and has a molecular weight range of 150–220 kDa [3]. The ratio and structure of 7S has been suggested to affect the functionality and physical properties of soy proteins [4,5,6]. Lovati et al. [7] confirmed that the 7S fraction was the major protein source in hyperglycemia mice. Furthermore, Marcello et al. [8] demonstrated that the α’ chain in 7S plays a key role in reducing blood fat by up-regulation of liver β-VLDL receptors. Additionally, increasing the 7S content progressively could improve gel water holding capacity. Molian et al. [9] found that 7S showed the highest emulsifying activity index and surface hydrophobicity after treatment by high pressure at 400 MPa. According to a recent report [10], blanching soybeans in the range of 60 to 80 °C promotes the texture of soymilk gel to be softer and much tender, which finally leads to the formation of 7S aggregation.

As a glycoprotein, 7S shows high affinity towards fatty acids, phospholipids and aromatic compounds, which have hydrophobic and amphiphilic properties [11]. Generally, soy proteins exist in both natural foods and many processed foods, such as soymilk beverages and douchi. In recent years, fruit juice-soymilk as a functional beverage has received increasing interest from industry beverage suppliers [12,13]. This complex juice not only masks the soymilk beany flavor, but also improves the nutritional properties of fruit juice-soymilk [14,15]. The effect of soy protein on the antioxidant activity of phenolic acids has been reported. Moreover, soy protein isolate (SPI) as an effective food-based delivery vehicle also exhibits strong anti-diabetic activity [16]. The protein-phenolic acids complexes may protect phenolic acids from oxidation through the gastrointestinal tract during their passage [17]. However, phenolic acid can influence the property of soy protein. Phenolic acids have been found to affect the solubility, stability and digestibility of proteins [18]. Phenolic acids can cross-link with 11S and affect the solubility of phenolic acids-soy protein complexes by changing the net charge of proteins [19]. Rawel et al. [20] revealed that phenolic acids may affect the availability of proteins by reducing the content of lysine and tryptophan residues. Chlorogenic acid may decrease the digestibility of whey proteins according to [21]. The addition of soy-protein-chlorogenic acid may improve the excretion of fecal and urinary nitrogen in rats [22]. However, currently, all reported studies on the interaction between phenolic acids and soy proteins have focused solely on 11S. There is no available literature reporting the interaction between different phenolic acids and 7S.

**Figure 1:**
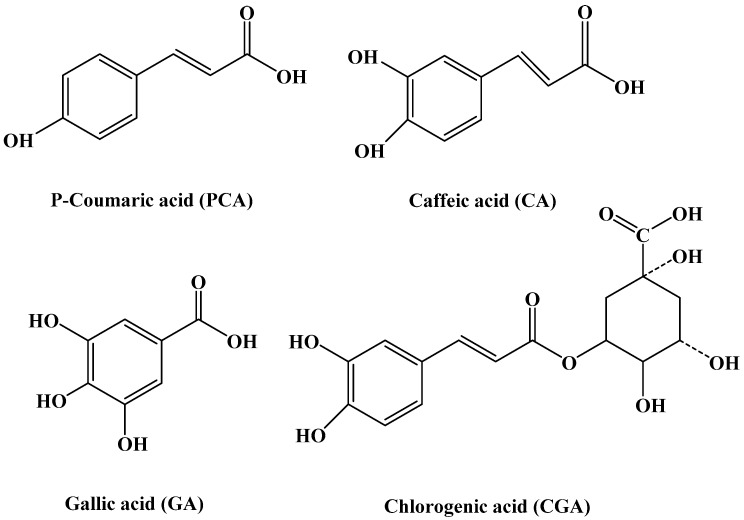
Chemical structures of the four phenolic acids (PCA, CA, GA and CGA).

Phenolic acids have been used to prevent the onset of chronic diseases because of their various positive bioactivities, including anti-oxidation, anti-carcinogenic properties and potential health promoting effects [23,24,25,26,27]. Phenolic acids have different structures, and can be distinguished from each other by the number and position of the hydroxyl groups on the aromatic ring. *p*-Coumaric acid (PCA), caffeic acid (CA), gallic acid (GA) and chlorogenic acid (CGA) are the most common phenolic acids found in fruit juices. PCA is defined as a single hydroxycinnamic acid, whereas CA is defined as a double hydroxycinnamic acid. GA is a triple hydroxyl benzoic acid. CGA is an ester of CA and quinic acid (Figure 1).

It is well known that soy proteins can be degraded by pepsin into peptides and amino acids, which could increase bio-accessibility and promote bioavailability, such as emulsifying properties, ACE (angiotensin converting enzyme)-activity and the bio-utilization of calcium [28]. Li et al. [29] found that the complex of tannic acid and soy ferritin could inhibit ferritin from degradation by proteases in vitro. Proanthocyanidins combined with soy ferritin inhibited protein degradation by pepsin and trypsin in vitro [30,31]. Thus, analyzing the degradation rates of 7S-phenolic acid complexes by pepsin and trypsin is of industrial interest.

In this report, the interaction between different phenolic acids (PCA, CA, GA and CGA) and soy protein 7S was examined by SDS-PAGE, circular dichroism (CD) spectra, fluorescence spectroscopy and dynamic light scattering (DLS). In addition, the influence of the number and position of the hydroxyl substituents of different phenolic acids on the structure of soy protein 7S was studied. The results of the digestion of 7S by enzymes of the gastro-intestinal tract (i.e., pepsin and trypsin) in the presence of phenolic acids are presented.

## 2. Results and Discussion

### 2.1. Purification and Characterization of Soy Protein 7S

**Figure 2:**
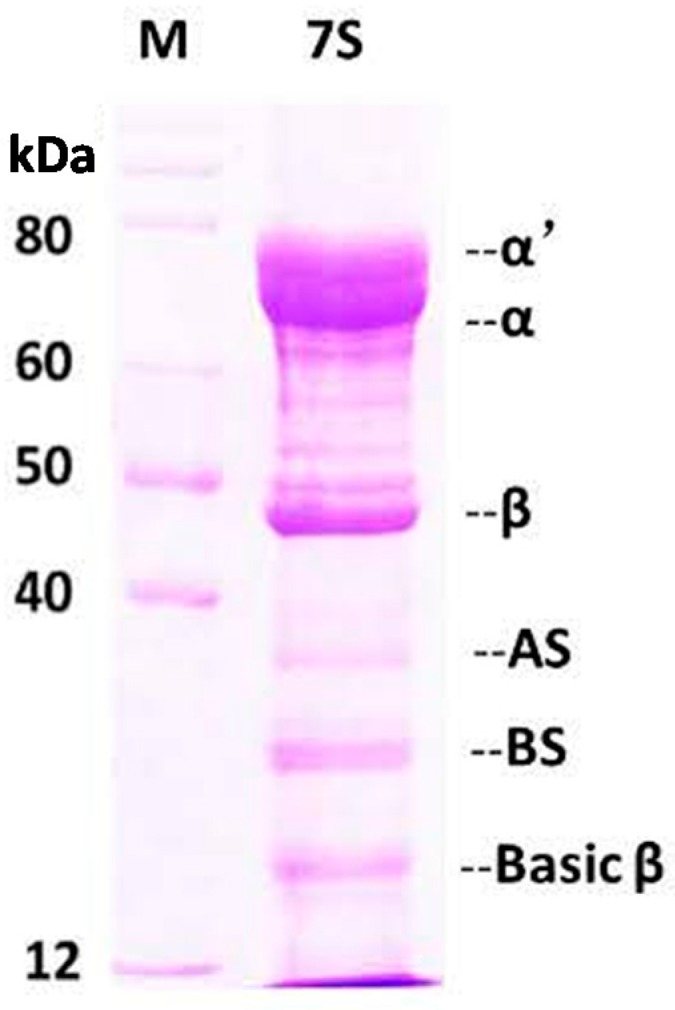
SDS-PAGE analysis of soy protein 7S. Conditions: 0.08 μM soy protein 7S in 100 mM 3-(N-morpholino) propanesulphonic acid(MOPS) (5 mM NaCl), pH 7.0, 25 °C.

Soy protein 7S was purified according to a reported method with minor modifications [32]. SDS-PAGE was used to visualize the subunit distribution of the protein (Figure 2). Gel images were digitalized and analyzed by densitometry with an alpha Imager 6.0 scanner (Alpha Innotech, San Leandro, CA, USA) to quantify the bands and level of purity. As shown by the gels, the soy protein 7S was the main fraction with subunits α (67 kDa), α’ (71 kDa), β (45–50 kDa) [5], observed. Soy protein 11S, with AS (32 kDa) and BS (28 kDa) subunits was also observed as a minor fraction. The purity of soy protein 7S was ~80%, as calculated by the Imager 6.0 scanner.

### 2.2. Interaction Between Different Phenolic Acids and Soy Protein 7S

A combination of SDS-PAGE, CD spectra, fluorescence spectroscopy and DLS was used to determine whether soy protein 7S interacts with different phenolic acids at pH 7.0. Treatment with PCA, CA, GA and CGA did not change the electrophoretic behavior of soy protein 7S, as observed by native-PAGE and SDS-PAGE (data not shown), indicating no alteration in the primary structure of soy protein 7S.

**Figure 3:**
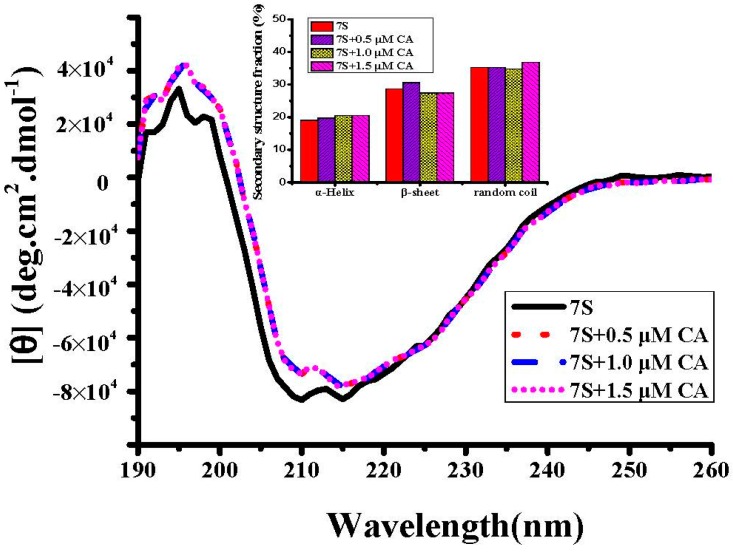
CD spectra of soy protein 7S in the presence and absence of CA. Inset: Estimated secondary structure fractions of soy protein 7S. Conditions: 0.5 μM soy protein 7S in 100 mM MOPS (5 mM NaCl), pH 7.0, 25 °C.

To analyze the effects of these phenolic acids on the secondary structure of soy protein 7S, CD experiments were recorded in the far-UV region (Figure 3). Native soy protein 7S has a single negative ellipticity at 208 nm, which is characteristic of a protein with a typical β-sheet secondary structure [33]. Soy protein 7S contains about 19% α-helix, 28.7% β-sheet and 35.3% random coil, as estimated by the program CDNN (Applied Photophysics, Leatherhead, UK). Treatment with CA did not alter significantly the secondary structure of soy protein 7S tent (Figure 3). Similarly, addition of PCA, GA and CGA had little effect on the protein secondary structure (date not shown). These results indicate that treatment of soy protein 7S with phenolic acids does not cause a change in the protein secondary structure. A similar observation was made when soy glycinin was treated with phenolic acids [19]. These results suggest that the second structure of soy protein 7S is not disrupted or altered by phenolic acids.

**Figure 4:**
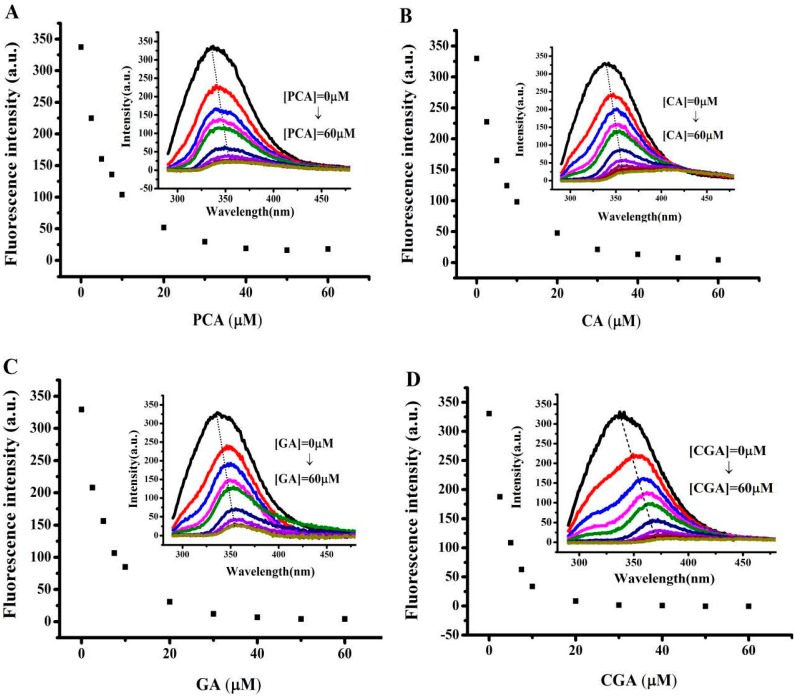
Fluorescence spectra of soy protein 7S and soy protein 7S treated with: (**A**) PCA; (**B**) CA; (**C**) GA; and (**D**) CGA at different concentrations. Conditions: 1 μM 7S in 100 mM MOPS (5 mM NaCl), [phenolic acids] = 60 μM, pH 7.0, 25 °C. *λ_E_*_x_ = 280 nm, slits for excitation and emission are 5 nm and 10 nm, respectively.

Fluorescence spectroscopy is widely used to study the structure and dynamics of proteins because most proteins emit intrinsic fluorescence. The fluorescence spectra of soy protein 7S in the absence and presence of different concentrations of the four phenolic acids in MOPS (pH 7.0) are shown in Figure 4. Soy protein 7S exhibited a strong fluorescence peak at 336 nm on excitation at 280 nm, indicating that most of the observed fluorescence is contributed by Trp residues. In contrast, soy protein 7S treated with phenolic acids exhibited different fluorescence intensities in the 415–425 nm region. With increasing concentration of the phenolic acids from 0 to 60 μM, the fluorescence intensity of soy protein 7S decreased, with a slight red shift of the maximum emission wavelength (Figure 4), indicating that the microenvironment around Trp was altered. When the concentration of PCA, CA, GA and CGA reached 60 μM, 90%, 93%, 95% and 91% of the protein fluorescence was quenched, respectively. This observation indicated that a strong interaction existed between each of the phenolic acids and soy protein 7S. The fluorophore can be quenched either by collision or by complex formation with the same quencher [17], leading to an alteration to the tertiary structure of 7S in the vicinity of Trp residues. Thus, the microenvironment around particular Trp residues changes from hydrophobic to hydrophilic because of the addition of PCA, CA, GA and CGA [34]. The above experiments were also carried out at pH 4.0 and similar results were obtained, as presented in supplementary materials (Figure S1).

**Figure 5:**
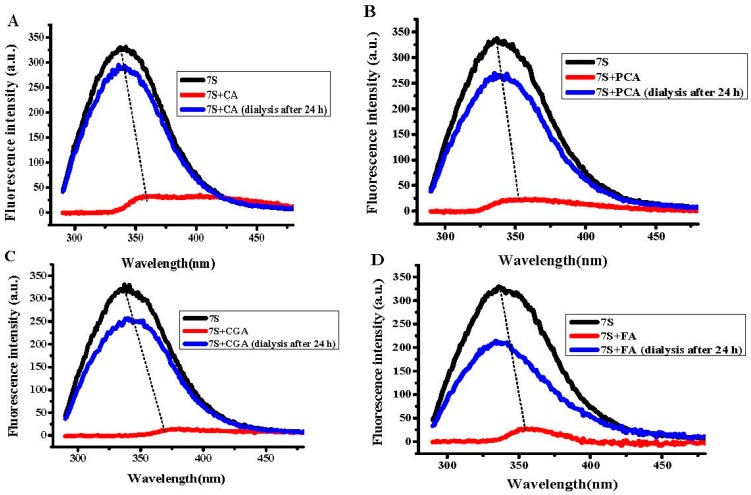
Fluorescence intensity of soy protein 7S treated with: (**A**) PCA; (**B**) CA; (**C**) GA; and (**D**) CGA after dialysis for 24 h. Conditions: 1 μM soy protein 7S in 100 mM MOPS (5 mM NaCl), [PCA, CA, GA or CGA] = 100 μM, pH 7.0, 25 °C. *λ*_Ex_ = 280 nm, slits for excitation and emission are 5 and 10 nm, respectively.

To determine whether the above observed interaction between phenolic acids and soy protein 7S is a result of the binding of these small molecules to the protein, dialysis experiments were carried out. Results were shown in Figure 5. Generally, all the fluorescence spectra of soy protein 7S was significant distinct from those of untreated protein sample. The maximal fluorescence emission intensity of soy protein 7S decreased by 52%, 73%, 74.5% and 55%, respectively. These results indicated that all of these phenolic acids can bind with 7S, while CA and GA molecules bind to 7S more tightly than PCA and CGA. Comparison of the structures of these four phenolic acids shows that CA has one more hydroxyl group than PCA located at the ortho-position of the benzene ring, and GA has one more hydroxyl group than CA (Figure 1). The hydroxylation on the ring A of phenolic acids significantly improved the affinities toward proteins [35,36]. Therefore, an increase of one hydroxyl group resulted in a large difference in protein fluorescence quenching between CA and PCA. However, CGA has three more hydroxyl groups located on the ortho-benzene ring, but showed a lower quenching effect than CA (Figure 5C). This observation may be a result of steric effects, where the binding site is not sufficiently large enough to accommodate the larger phenolic acid moiety. These results suggest that the 7S-binding activity of these phenolic acids compounds is structure-dependent.

**Figure 6:**
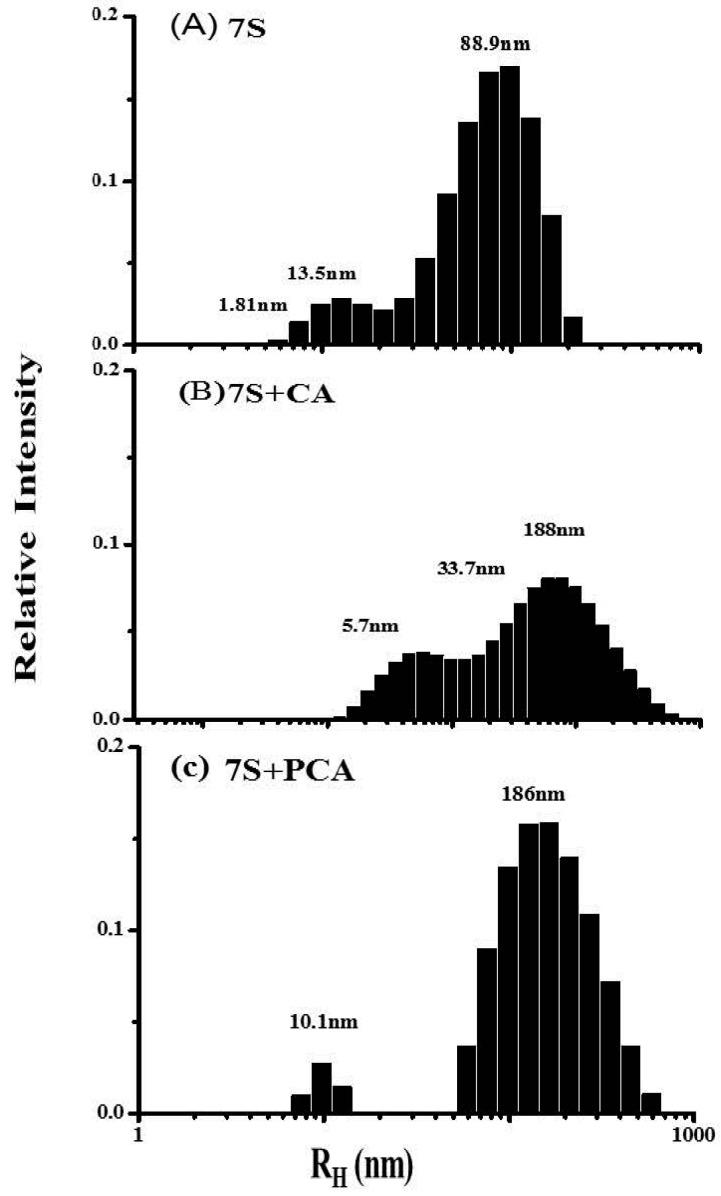
Relative scattered light intensity distribution curves for 0.5 μM soy protein 7S (**A**); and 0.5 μM soy protein 7S in the presence of 5 mM CA (**B**); or 5 mM PCA (**C**). Conditions: 100 mM MOPS (5 mM NaCl), pH 7.0, 25 °C.

The fluorescence results indicated that there is an interaction between phenolic acids and soy protein 7S that alters the tertiary structure of soy protein 7S in the vicinity of Trp residues. Subsequently, we used DLS to investigate the consequences of such interactions (Figure 6). Three populations with *R*_H_ values of 1.81, 13.5 and 88.9 nm are present in the scattered light intensity distribution curve of the soy protein 7S sample (Figure 6A). The population centered at 88.9 nm was the largest with ~80% of the total signal, whereas the second population had an *R*_H_ = 13.5 nm and the third population was 1.81 nm. After addition of CA and PCA to soy protein 7S, the size distribution changes toward large aggregates (Figure 6B,C), indicating that soy protein 7S self-associates. Similarly, addition of GA and CGA also had a strong effect on protein size (data not shown). These findings confirmed the conclusion that phenolic acids were able to facilitate soy protein 7S association. Several researchers have found that phenolic acids have strong affinity with salivary proteins, glycinin and phytoferritin, which are rich in proline [37,38]. The hydroxyl groups on phenolic acids easily form strong hydrogen bonds with the amide carbonyl of the peptide backbone [39,40]. In addition, hydrophobic interactions between the phenolic acids and proteins were observed [41]. Thus, it is reasonable to postulate that the interaction between phenolic acids and soy protein 7S may be a result of both hydrogen bonds and hydrophobic interactions.

### 2.3. Effect of Different Phenolic Acids on the Digestive Stability of Soy Protein 7S

**Figure 7:**
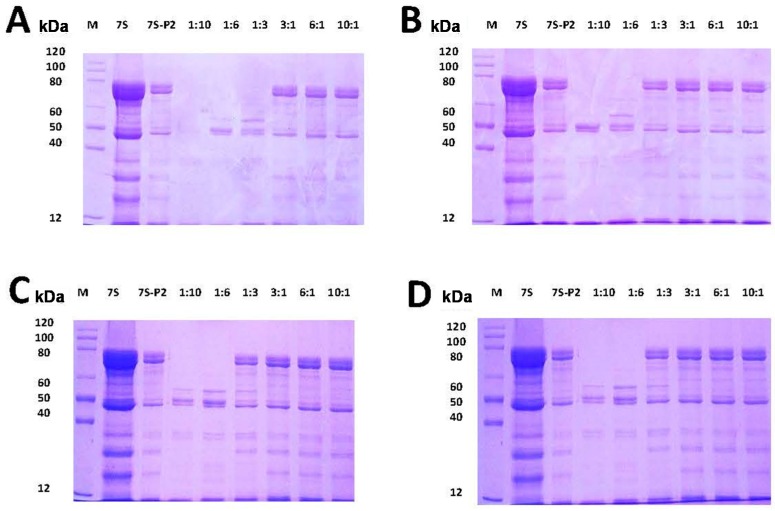
SDS-PAGE analysis of the digestive stability of soy protein 7S in the absence and presence of: PCA (**A**); CA (**B**); GA (**C**); and CGA (**D**) at different concentrations in simulated gastric fluid (pH 2.0). Conditions: [soy protein 7S] = 3 μM; the ratios of protein to phenolic acid: 1:10, 1:6, 1:3, 3:1, 6:1 and 10:1. “7S-P2” represents a mixture of pepsin and soy protein 7S in the absence of phenolic acids.

**Figure 8:**
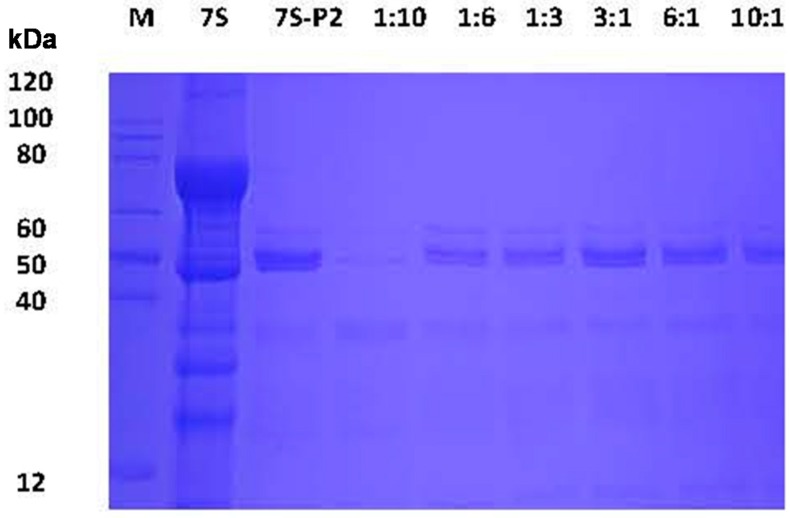
SDS-PAGE analyses of the digestive stability of soy protein 7S in the absence and presence of PCA at different concentrations in simulated intestinal fluid (pH 7.5). Conditions: [soy protein 7S] = 3 μM; the ratio of protein to phenolic acid: 1:10, 1:6, 1:3, 3:1, 6:1 and 10:1. “7S-P2” represents a mixture of trypsin and soy protein 7S in the absence of phenolic acids.

In general, protein digestion by proteases is dependent on the structure of a protein and the environment surrounding the protein [21]. Changes in the environment by the addition of compounds may lead to changes in the tertiary structure of proteins because of direct interaction of the added compound(s) with the protein [19]. Studies have shown that protein-tannin aggregates decrease the digestion of proteins in vitro. Therefore, understanding whether such interactions can affect the stability of soy protein 7S against degradation by pepsin and trypsin is of interest. To test soy protein 7S protease resistance, this protein was mixed with PCA, CA, GA and CGA with different mass ratios (1:10, 1:6, 1:3, 3:1, 6:1, 10:1) at pH 2.0 and pH 4.0, and the mixtures were digested for 1 h at 37 °C and subsequently analyzed by SDS-PAGE (Figure 7). At pH 2.0, soy protein 7S was degraded into peptides by pepsin, as shown in the lane termed “7S-P2”. Treatment with PCA, CA, GA and CGA did not inhibit soy protein 7S degradation by pepsin, even when the mass ratio of phenolic acids to soy protein 7S reached 10:1, suggesting that these four phenolic acids do not inhibit soy protein 7S degradation by pepsin at pH 2.0, a condition similar to that of the adult simulated gastric fluid. Subsequently, the stability of 7S in the absence and presence of PCA, CA, GA and CGA was determined by SDS-PAGE in simulated trypsin fluid at pH 7.5. The result of 7S-PCA aggregates digested by trypsin is shown in Figure 8. Clearly, 7S was digested by trypsin in the sample (lane 2) and PCA did not affect degradation by trypsin, even when the mass ratio ranged between 1:10 and 10:1. Similarly, addition of CA, GA and CGA did not inhibit the degradation of 7S by trypsin (data not shown).

In parallel experiments, the stabilities of 7S in the absence and presence of the four phenolic acids were also investigated in a simulated stomach environment at pH 4.0 (Figure S2). The phenolic acids did not prevent the degradation of soy protein 7S by pepsin. This is most likely because the binding sites of phenolic acids to soybean protein 7S do not overlap with the hydrolyzed sites of the protein by pepsin. Moreover, we found that addition of phenolic acids did not caused pepsin precipitation under all tested conditions.

## 3. Materials and Methods

### 3.1. Chemicals

*p*-Coumaric acid (PCA), caffeic acid (CA), gallic acid (GA) and chlorogenic acid (CGA) with a purity of 98% were purchased from Sigma-Aldrich Co. LLC. (St. Louis, MO, USA). Pepsin and trypsin were purchased from J&K Chemical (Beijing, China). All other reagents used were of analytical grade or purer (BioDee Bio Tech Corporation Led, Beijing, China).

### 3.2. Preparation of Soy Protein 7S

Soy protein 7S was extracted according to a reported method with some modifications [42], and the procedure was as follows: 100 g protein meal was added to 1.5 L distilled water, and the pH value of the suspension was adjusted to 8.2–8.5 with 2 mol/L NaOH. The mixture was then stirred at room temperature for 1.5 h, followed by filtration with 120-mesh sieve and centrifuged at 8000× *g* for 20 min. Then, 15 g sodium hydrogen sulfite (SBS) was added to the supernatant and the pH was set to 5.4 with 2 mol/L HCl. After 3 h, the resulting solutions were centrifuged at 6000× *g* for 20 min, and the pH of the resulting supernatant was adjusted to 4.5 with 2 mol/L HCl, and NaCl was added until the ionic strength reached to 0.25 mol/L. The resulting solution was placed at –4 °C for 10 h and then centrifuged at 6000× *g* for 20 min at 4 °C. The precipitate was soy protein 11S. The concentrations of soy protein 7S were determined according to the Bradford (1976) with bovine serum albumin (BSA) as the standard. The purity of the 7S samples was examined by native- and SDS-PAGE.

### 3.3. Fluorescence Titration

Fluorescence titration experiments were executed by a Cary Eclipse spectrophotometer (Varian, Polo Alto, CA, USA). The concentration of soy protein 7S was 1 μM in 100 mM MOPS, pH 7.0, at 25 °C. Additionally, 1 μM soy protein 7S was dissolved in sodium acetate-acetic acid buffer, pH 4.0, at 25 °C. The titrations were measured by adding 2 μL of PCA, CA, GA and CGA to 2 mL soy protein 7S according to the reported method [29] with some modifications. Fluorescence spectra were scanned from 290 to 450 nm after each addition. The excitation wavelength was set to 280 mm. Fluorescent spectra of the individual phenolic acids were also recorded under the same conditions and the phenolic acid signal was subtracted from the signal arising from the corresponding phenolic acid-soy protein 7S complex.

The dialysis experiments were carried out as followed: 2 mL of soy protein 7S (1 μM) was incubated with 10 μL PCA, 10 μL CA, 10 μL GA and 10 μL CGA (10 mM), respectively, and then the mixture was dialyzed in 0.1 M 3-(N-morpholino) propanesulphonic acid(MOPS) buffer with 50 mM NaCl to remove free phenolic acids. After 24 h, the fluorescence spectra of soy protein 7S were detected.

The fluorescence emission intensity of soy protein 7S was defined as the initial fluorescence intensity (A). Phenolic acids (PCA, CA, GA and CGA) were added to soy protein 7S, respectively, until the fluorescence intensity stabilized. This fluorescence intensity was considered to be the maximum fluorescence emission intensity (B). The decreased ratio of fluorescence emission intensity (C) was determined as:

C = (A − B)/A × 100%

### 3.4. Circular Dichroism (CD) Spectra

As previous described [30], protein samples were dissolved in 100 mM MOPS buffer, pH 7.0. CD spectra were recorded with a PiStar-180 spectrometer (Applied Photophysics, Leatherhead, UK) using quartz cuvettes of 1 mm optical path length at 25 °C. CD spectra were measured in the far UV range (190–260 nm) with 5 replicates at 50 nm/min and a bandwidth of 1 nm. The CD data were expressed in terms of mean residual ellipticity (θ), in deg·cm^2^·dmol^–1^. The change in protein ellipticity induced by treatment with PCA, CA, CG and CGA was determined as the ellipticity of the phenolic acid-protein mixture minus the CD signal arising from a solution of only PCA, CA, GA, or CGA under the same experimental conditions. K2D2 was used to calculate the percentage of secondary structure.

### 3.5. Dynamic Light-Scattering (DLS) Experiments

The DLS measurements were carried out at 25 °C by a Viscotek model 802 dynamic light-scattering instrument (Viscotek Europe Ltd., Berks, UK) and according to earlier reports [31]. Two-milliliter volumes of 7S (0.5 μM dissolved in 100 μM MOPS, pH 7.0) and 10 μL phenolic acids (5 mM) were mixed at 25 °C. Each measurement was averaged over 20 runs. The size of the protein sample was calculated as the hydrodynamic radius (*R*_H_) using the sphere model. Using the *R*_H_ values, estimates of the relative molecular weights of the samples were determined based on a standard curve calibrated with known proteins. The size of the complex was evaluated by the OmniSIZE 2.0 software (Viscotek Europe Ltd.).

### 3.6. Simulated Gastric Fluid and Simulated Intestinal Fluid Digestion Stability Assay

The simulated gastric fluid digestion experiment was performed according to a recent study [19]. All samples were digested in two simulated stomach models (pH = 2.0 or 4.0) in vitro. Briefly, 200 μL of simulated gastric fluid mix with 40 μL of 7S protein in the presence of various phenolic acids (0–10 mg/mL) was reacted at 37 °C. After 1 h, 60 μL of 1 M NaOH was injected to terminate the reaction. The simulated intestinal fluid experiments were carried out according to United States Pharmacopoeia [43]. The simulated intestinal fluid (SIF) consisted of 10 mg/mL trypsin, which was dissolved in 100 mM KH_2_PO_4_-NaOH mixing buffer at pH 7.5. Two hundred microliters of the SIF mix with 40 μL of the 7S protein in the presence of various phenolic acids (0–10 mg/mL) was reacted at 37 °C. After 1 h, the reaction was immediately terminated by placing the sample in boiling water for 10 min. Subsequently, samples were analyzed by SDS-PAGE.

### 3.7. Statistical Analysis

All data analysis was performed using the Origin 8.0 software (OriginLab, Northampton, MA, USA) and structural formulas were processed by ChemDraw 7.0 (CambridgeSoft, Cambridge, MA, USA). All experiments were carried out at least twice.

## 4. Conclusions

In the present work, SDS-PAGE, CD, fluorescence spectroscopy and DLS were used to analyze the interaction between a soy protein and phenolic acids (PCA, CA, GA and PCA). The results from the experiments showed that the phenolic acids examined interact with soy protein 7S in a structure-dependent manner. The number of hydroxyl groups on the aromatic ring moiety of the phenolic acids determined the affinity towards soy protein 7S. Interestingly, protein-phenolic acid aggregates did not affect the digestion of soy protein 7S by pepsin or trypsin at either pH 2.0 or pH 4.0. This is the first report to clarify the interaction between phenolic acids and soybean protein 7S, as well as its influence on the function of proteins. These findings should favor the use of soy protein-based foods.

## Supplementary Materials

Supplementary materials can be found at http://www.mdpi.com/1422-0067/17/10/1671/s1.

## Abbreviations

PCA	*p*-Coumalic Acid
CA	Caffeic
GA	Gallic Acid
CGA	Chlorogenic Acid
7S	β-Conglycinin
CD	Circular Dichroism
DLS	Dynamic Light-Scattering
