# Supplementary material for: Interactions of β-Conglycinin (7S) with Different Phenolic Acids—Impact on Structural Characteristics and Proteolytic Degradation of Proteins
Source: Int J Mol Sci. 2016 Oct 2;17(10):1671. doi: 10.3390/ijms17101671 (PMC5085704; doi:10.3390/ijms17101671)
Supplement: Supplementary file 1 [file ijms-17-01671-s001.pdf]

Supplementary Materials: Interactions of β-Conglycinin (7S) with Different Phenolic Acids—Impact on Structural Characteristics and Proteolytic Degradation of Proteins

Jing Gan, Hao Chen, Jiyuan Liu, Yongquan Wang, Satoru Nirasawa and Yongqiang Cheng

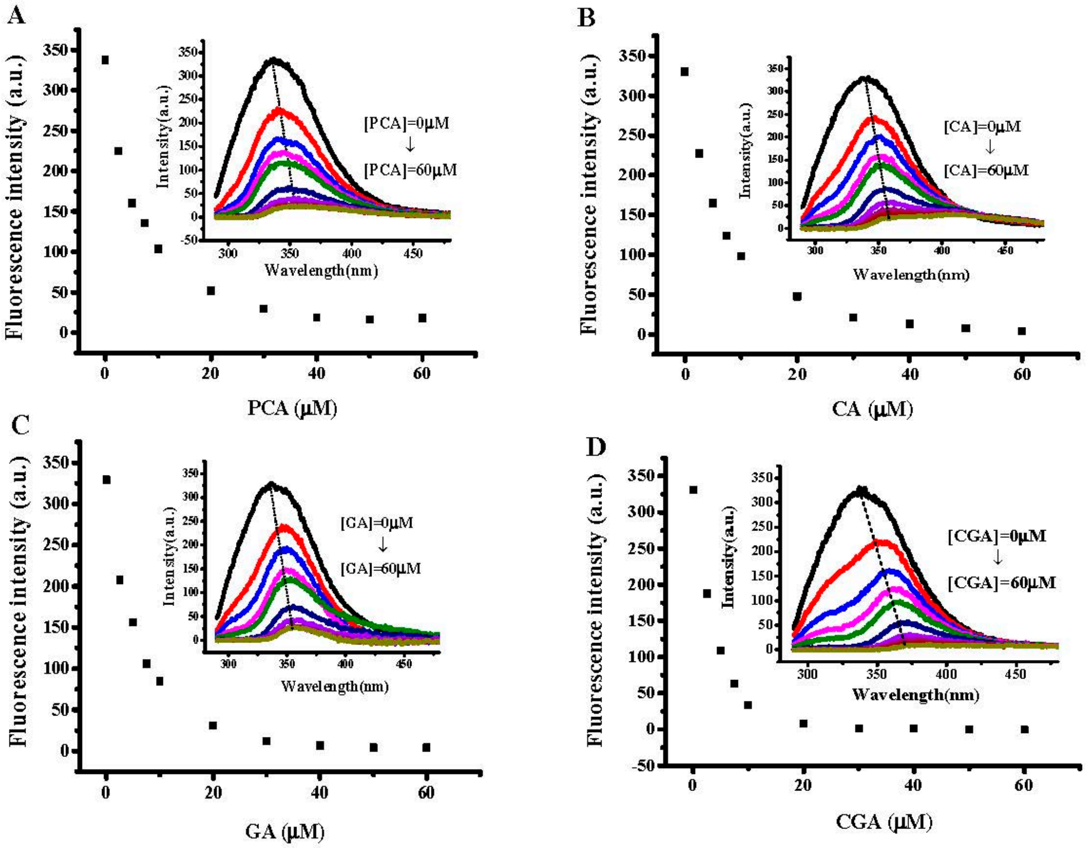

Figure S1. Fluorescence spectra of soy protein 7S and soy protein 7S treated with (**A**) PCA; (**B**) CA; (**C**) GA; and (**D**) CGA at different concentrations, respectively. Conditions: 1 μM 7S in 10 mM MOPS, 5 mM NaCl, [phenolic acids] = 60 μM, pH 4.0, 25 °C. λ_{Ex} =280 nm, slits for excitation and emission are 5 nm and 10 nm.

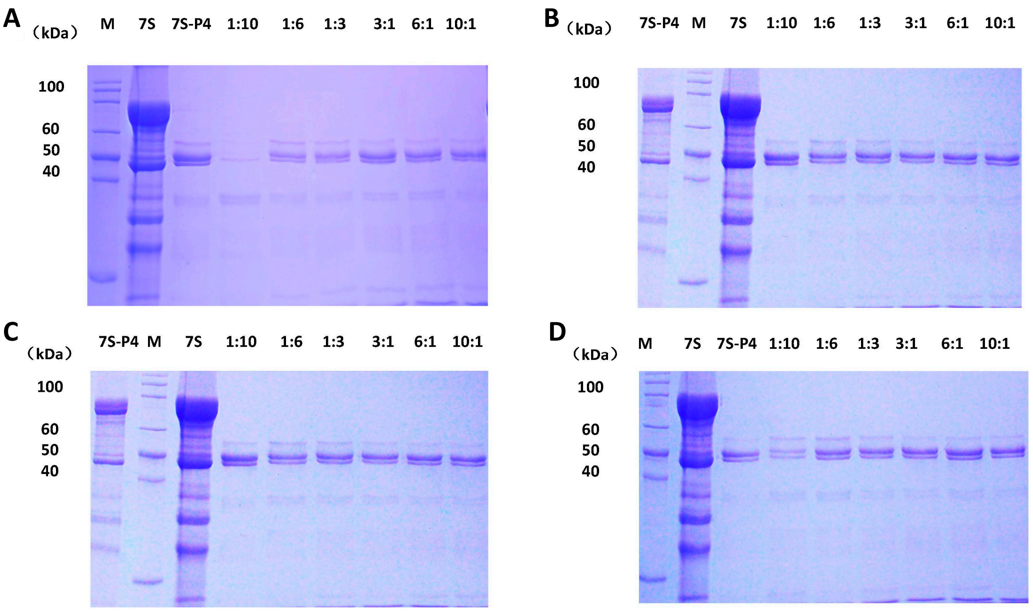

Figure S2. SDS-PAGE analyses of the digestive stability of soy protein 7S in the absence and presence of PCA (**A**); CA (**B**); GA (**C**); and CGA (**D**) at different concentrations in simulated gastric fluid (pH 4.0). Conditions: [soy protein 7S] = 3 μM, the ratio of protein to phenolic acid is over the range of 1:10, 1:6, 1:3, 3:1, 6:1, and 10:1. "7S-P4" represents a mixture of pepsin and soy protein 7S in the absence of phenolic acids.
